# Examination of the Effect of a 50-Hz Electromagnetic Field at 500 μT on Parameters Related With the Cardiovascular System in Rats

**DOI:** 10.3389/fpubh.2020.00087

**Published:** 2020-04-07

**Authors:** Yemao Zhang, Lijuan Li, Xingfa Liu, Lijian Ding, Xiong Wu, Jin Wang, Mengying He, Huiying Hou, Guoran Ruan, Jinsheng Lai, Chen Chen

**Affiliations:** ^1^School of Electrical Engineering and Automation, Hefei University of Technology, Hefei, China; ^2^State Key Laboratory of Power Grid Environmental Protection, High Voltage Research Institute, China Electric Power Research Institute, Wuhan, China; ^3^Division of Cardiology and Hubei Key Laboratory of Genetics and Molecular Mechanisms of Cardiological Disorders, Tongji Hospital, Tongji Medical College, Huazhong University of Science and Technology, Wuhan, China

**Keywords:** electromagnetic field, cardiovascular system, physiology, cardiac remodeling, rat

## Abstract

**Background:** Whether electromagnetic field (EMF) exposure affects the function of the cardiovascular system is under debate. The present study aimed to investigate the effects of 500 μT EMF exposure on the cardiovascular system in rats.

**Methods:** Forty-eight-week-old male Sprague-Dawley rats were randomly divided into two groups: the sham group and the exposure group. During 24-week EMF exposure (20 h per day), the blood pressure and pulse rate were recorded every 4 weeks. Before sacrifice, electrocardiography, echocardiography, and cardiac catheterization analysis were conducted to evaluate the cardiac function. Meanwhile, hematoxylin-eosin (HE) staining, Western blot, and real-time polymerase chain reaction (PCR) were performed to identify morphological and molecular changes indicative of cardiac remodeling.

**Results:** The heart rate, blood pressure, and pulse rate were not influenced by EMF exposure compared with the control group. In addition, HE staining showed no change in the morphology and arrangement of cardiomyocytes. Further, we found that the mRNA and protein levels of cardiac hypertrophy-related genes were not affected by EMF exposure. Finally, no significant difference was observed in cardiac function between the two groups by echocardiography and cardiac catheterization detection.

**Conclusion:** The 24-week exposure to EMF at 500 μT did not have apparent effects on the cardiovascular system in rats, at least for the variables studied.

## Introduction

In past decades, with the development of science and technology, many electrical devices and equipment that can generate electromagnetic fields (EMFs) have brought great convenience to our daily life. At the same time, the probability of exposure to these EMFs is increasing dramatically (1). It is well-accepted that EMFs can be generated by a variety of appliances, such as transformers, electrical panels, televisions, and electric blankets (2, 3), whose frequency is usually 50- or 60-Hz (4). Human-made EMFs, such as nonionizing radiation, are classified into three categories: extremely low-frequency fields (<300-Hz), high-frequency fields in the radio-frequency band (300-Hz to 3 × 10^8^ Hz), and microwaves [3 × 10^8^ to 3 × 10^11^ Hz; (5–7)]. Thus, the effects of 50-Hz EMF exposure on human health have attracted more and more attention. Crasson et al. (8) suggested that exposure to a 50-Hz EMF at 100 μT for 3 weeks (30 min per day) had no effect on the level of plasma melatonin in healthy male volunteers. Touitou et al. (9) found that long-term exposure (up to 20 years) to a 50-Hz EMF did not affect the hematological and immune system functions in healthy men. In contrast, Brendel et al. (10) exposed isolated pineal glands of Djungarian hamsters to a 1,623- or 50-Hz EMF at 86 μT for 8 h and found that EMF exposure could suppress melatonin synthesis in the pineal gland *in vitro*. Most studies on the effects of EMF have been focused on cancer. Some studies hold the view that 50-Hz EMF exposure might promote the initiation and progression of cancer. Thun-Battersby et al. (11) revealed that exposure to a 50-Hz EMF at 100 μT for 27 weeks (24 h per day) accelerated the growth of mammary tumor in DMBA-treated breast cancer rat. Similar results were also observed by Mevissen et al. (12) who exposed DMBA-treated breast cancer rats to a 50-Hz EMF at 50 μT for 91 days (24 h per day), and Rannug et al. (13) who employed intermittent exposure of 50-Hz at 50 and 0.5 μT, respectively, for 104 weeks (19–21 h per day) on SENCAR skin tumor mice (13). However, there were also studies showing that lifespan exposure to a continuous or intermittent 50-Hz EMF alone did not significantly increase oncogenesis in rats (14).

The cardiovascular system is mainly made up of arteries, veins, and capillaries. Its function is complicated, mainly transporting the blood throughout the body to meet the demands of the body for oxygen and nutrients, which plays a key role in maintaining homeostasis in organisms. The cardiovascular system is important to human health, and its dysfunction will lead to various cardiovascular diseases, such as atherosclerosis (15), cardiac hypertrophy (16), heart failure (17), and so on.

To date, studies on the effects of EMFs on the cardiovascular system are limited. In 1999, Savitz et al. (18) investigated the mortality of cardiovascular diseases in 138,903 male electric utility workers from five American companies from 1950 to 1958, and the results showed that the duration of occupational EMF exposure was positively correlated with the incidence of arrhythmia and acute myocardial infarction. Recently, it was suggested that exposure to a 50-Hz EMF at 3 mT for 2 months (4 h per day) could induce oxidative stress and increase lipid peroxidation, resulting in apoptosis and morphological changes in the cardiomyocytes of adult rats (19). However, other studies did not observe any effects of EMF exposure on the cardiovascular system. Korpinen and Partanen (20, 21) first revealed that 1 h exposure to 50-Hz EMFs (1.4–6.6 μT) did not influence blood pressure or heart rate in human subjects. Whittington et al. (22) demonstrated that exposure to a 50-Hz EMF at 100 μT for 9 min did not affect cardiovascular performances in human subjects, as indicated by systolic blood pressure, diastolic blood pressure, mean arterial pressure, and heart rate. In 2016, our group found that exposure to a 50-Hz EMF at 100 μT for 24 weeks did not show any obvious effects on the cardiovascular system in Sprague-Dawley (SD) rats (23). Recently, we also found that 1 h continuous or 75 min intermittent exposure to a 50-HZ EMF at 100 μT caused no DNA damage in cardiomyocytes derived from both humans and rats (4).

We speculate that the above contradictory results are partially owing to the different designs of studies and detection methods. The potential impacts of EMF exposure on the cardiovascular system need further investigation. Therefore, different experiments with various examinations should be designed to test and verify the effects of EMF exposure on the cardiovascular system. Previously, we found that 100 μT EMF, which is the threshold of public exposure, did not affect the cardiovascular system and other organs (4, 23–26). In the current study, we elevated the intensity to 500 μT, which is the threshold of occupational exposure, to explore whether it would exert damages in the cardiovascular system in rats.

## Methods

### Ethics Statement

All animal experiments were performed in accordance with the ARRIVE guidelines and NIH guidelines for animal welfare. The protocol was approved by the Committee on the Ethics of Animal Experiments of the Animal Research Committee of Tongji College. Rats were fed in the Experimental Animal Center of Tongji Hospital, Tongji Medical College, Huazhong University of Science and Technology. We made all efforts to reduce the pain and distress of the animals, and the rats were euthanatized by CO_2_ inhalation before sacrifice.

### Animals

Forty-eight male Sprague-Dawley (SD) rats at the age of 8 weeks were randomly divided into two groups: the sham group (*n* = 24) and the exposure group (*n* = 24). The exposure group was exposed to a 50-Hz EMF at 500 μT for 24 weeks, 20 h per day, while the other group was sham-exposed. Animals were housed under standard laboratory conditions (12 h light and 12 h dark cycle, light cycle: 7:00–19:00 h with lights on at 07:00 a.m.; the room temperature was 23 ± 2°C, and the relative humidity was 50 ± 5%). The standard laboratory rodent diet and water were sterilized with high pressure, filtration, and ozone. The exposure system was exactly the same as described previously (Supplementary Figure 1) (23).

### Electrocardiography Analyses

After 24 weeks of exposure, the rats were anesthetized, and then the electrocardiography analyses were performed as described previously (23).

### Blood Pressure Measurements

The blood pressure measurements, including systolic pressure, diastolic pressure, and mean pressure and the pulse rate were recorded every 4 weeks by a Softron BP-98A system (Softron Biotechnology, Beijing, China), as described previously (23).

### Hematoxylin–Eosin (HE) Staining

After sacrifice, the hearts of rats were collected and fixed in 4% formalin for paraffin embedding. HE staining was then performed to assess the morphology and arrangement of cardiomyocytes. Images were obtained using the Nikon TE2000-U microscope (Nikon, Tokyo, Japan).

### Western Blot

Western blot was performed using the specific antibodies, as described previously (27). Antibody against BNP (Catalog No: A2179) was purchased from ABclonal Technology (Wuhan, China), and antibodies against GAPDH (Catalog No: 60004-1-Ig), ANP (Catalog No: 27426-1-AP), and Myh6 (Catalog No: 22281-1-AP) were obtained from Proteintech (Wuhan, China). Results were analyzed with Image J software (National Institutes of Health software).

### Quantitative Real-Time Polymerase Chain Reaction (PCR)

Total RNA was extracted from rat hearts with Trizol reagent (Invitrogen, Life Technologies, Carlsbad, CA) and reverse-transcribed into cDNA with a specific RT primer (TaKaRa, Dalian, China). Real-time PCR assays were performed using SYBR Green (Bimake, Shanghai, China) with specific primers on a 7900HT FAST real-time PCR system (Life Technologies, Carlsbad, CA). Data analysis was performed by the 2^−ΔΔ*Ct*^ method, as described previously (28). The primers are listed in Supplementary Table 1.

### Echocardiography and Hemodynamics Analyses

Before sacrifice, the rats were anesthetized, and then echocardiography analysis was performed to evaluate the cardiac functions using a high-resolution imaging system with a 30-MHz high-frequency scanhead (Vevo770, VisualSonics Inc, Toronto, Canada), as previously described (29). In order to measure the hemodynamics of the left ventricle, a pressure-volume catheter (Millar 1.4F, SPR835, Millar Instruments, Houston, TX) was inserted into the right carotid artery and through into the left ventricle to assess the intraventricular pressure and volume; the protocols were described previously (30).

### Statistical Analysis

All results are presented as mean ± standard deviation (S.D.). Independent samples *t*-test was performed to determine the statistical significance of differences between the two groups. A *p* < 0.05 was considered to indicate statistical significance.

## Results

### Exposure to a 50-Hz EMF at 500 μT Had No Effect on Cardiac Rhythm

**Figure 1:**
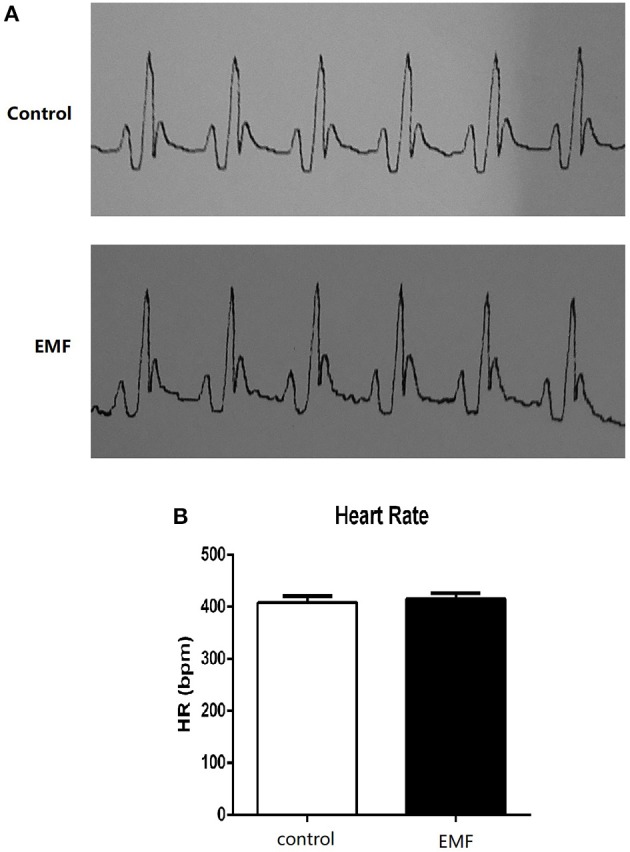
Exposure to a 50-Hz EMF at 500 μT had no effect on cardiac rhythm. **(A)** Electrocardiograms of rats from the two groups. **(B)** Heart rate (HR) of rats from the two groups.

The cardiac conduction system (CCS) comprises three main parts: the sinoatrial node, atrioventricular node, and Purkinje fibers. It can generate and conduct electrical impulses, consequently resulting in the contraction of the cardiomyocytes (31–33). In order to investigate the effects of 500 μT EMF on cardiac rhythm, the treated rats were subjected to electrocardiogram examination. As shown in Figure 1A, the electrocardiograms of rats from the two groups were similar, and no obvious arrhythmia was observed in either group. Meanwhile, the heart rate from the EMF group did not show significant change compared with the rats from the control group (Figure 1B).

These results indicated that exposure to a 50-Hz EMF at 500 μT had no effects on cardiac rhythm in rats.

### Exposure to a 50-Hz EMF at 500 μT Had No Effect on Blood Pressure

**Figure 2:**
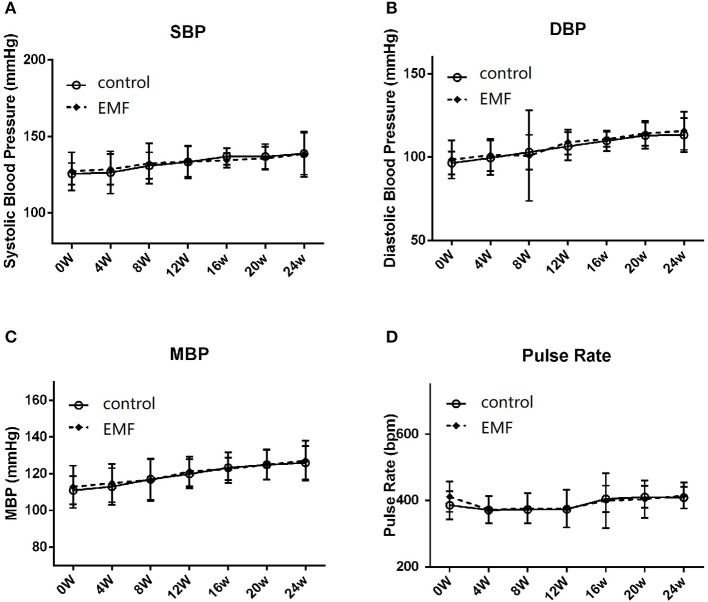
Exposure to a 50-Hz EMF at 500 μT had no effect on blood pressure. **(A)** Systolic blood pressure of rats from the two groups. **(B)** Diastolic blood pressure of rats from the two groups. **(C)** Mean blood pressure of rats from the two groups. **(D)** Pulse rate of rats from the two groups.

Blood pressure is a dynamic variable, like heart rate, that changes all the time. It is mainly reflected by systolic blood pressure and diastolic blood pressure (34). In the current study, the blood pressure and pulse rate of all rats were recorded every 4 weeks. The results showed that the systolic blood pressure, diastolic blood pressure, and mean blood pressure were not significantly different between the two groups (Figures 2A–C). Additionally, the pulse rate of the EMF exposure group was the same as that of the control group (Figure 2D).

These data suggested that exposure to a 50-Hz EMF at 500 μT did not affect the blood pressure in rats.

### Exposure to a 50-Hz EMF at 500 μT Had No Effect on Cardiac Hypertrophy

**Figure 3:**
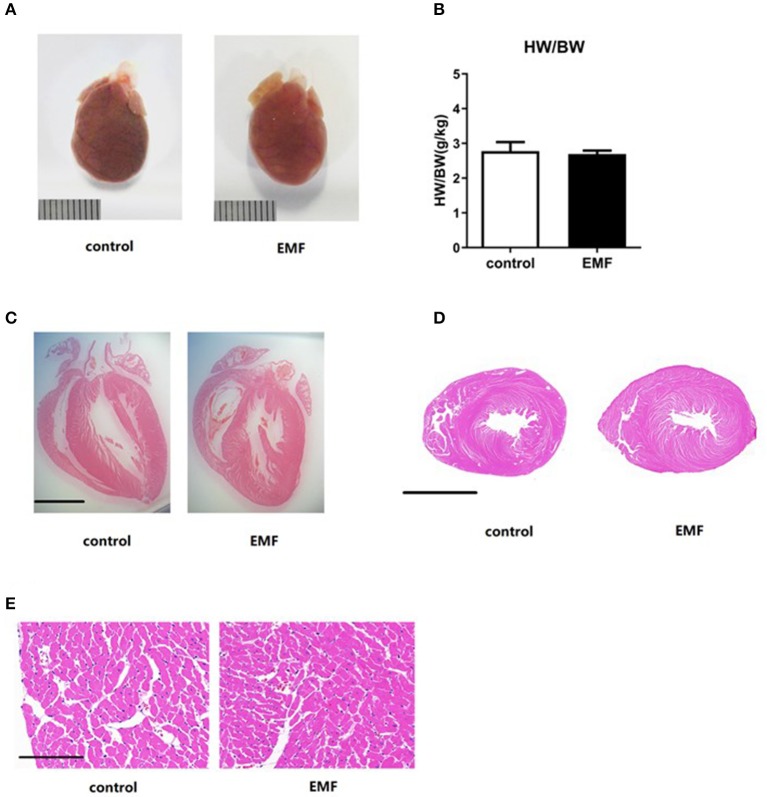
Exposure to a 50-Hz EMF at 500μT had no effect on cardiac hypertrophy. **(A)** Globe view of the hearts of rats from the two groups. Scale bar = 1 cm. **(B)** Ratio of heart weight to body weight of rats from the two groups. **(C,D)** Morphology of coronal and transverse sections of the heart of rats from the two groups. Scale bar = 5 mm. **(E)** Morphology of the cardiomyocytes of rats from the two groups. Scale bar = 150 μm.

Cardiac hypertrophy is a compensatory response to overload stress and is characterized by an increase in the heart mass, while sustained overload stress will lead to cardiac remodeling, finally resulting in heart failure (35–37). The rats were sacrificed after 24 weeks of exposure, and the hearts were collected. The results showed that the gross observations of the hearts from the two groups were the same (Figure 3A). There was no significant difference between the two groups in the ratio of heart weight to body weight (Figure 3B). Further, the morphology of coronal and transverse sections of the hearts showed no significant difference after 500 μT EMF exposure (Figures 3C,D). Moreover, HE staining indicated that EMF exposure had no significant effect on the morphology and arrangement of the cardiomyocytes compared with the sham exposure group (Figure 3E).

Together, the EMF exposure exerted no effect on cardiac hypertrophy in rats.

### Exposure to a 50-Hz EMF at 500 μT Had No Effect on the Expressions of Cardiac Remodeling-Related Genes

**Figure 4:**
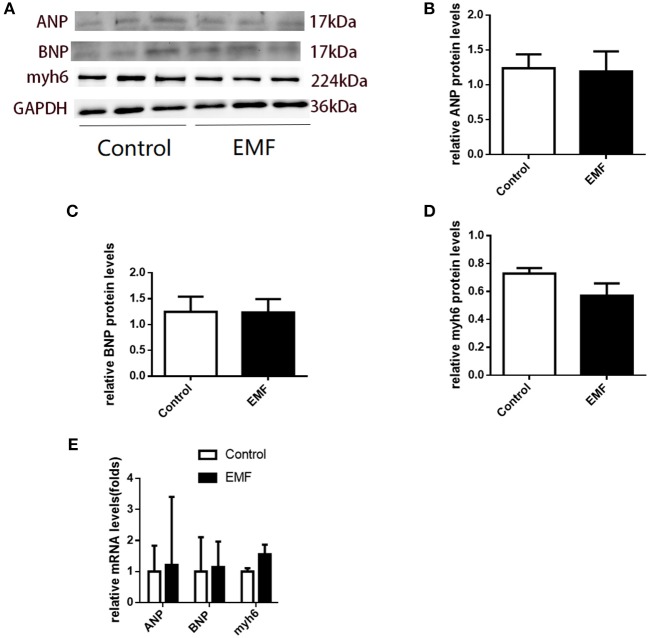
Exposure to a 50-Hz EMF at 500 μT had no effect on the expressions of cardiac remodeling-related genes. **(A)** Protein levels of cardiac remodeling-related genes in the heart of rats from the two groups. **(B–D)** Quantitative analysis of the protein levels of cardiac remodeling-related genes in the heart of rats from the groups. **(E)** mRNA levels of cardiac remodeling-related genes in the heart of rats from the two groups.

Although cardiac hypertrophy was not observed on the basis of morphology, cardiac remodeling can be induced at the molecular level, which would progress to visible changes in the future. In order to evaluate the expressions of the signals involved in cardiac remodeling, quantitative real-time PCR and Western blot assays were performed. No significant differences were observed in the expressions of ANP, BNP, and Myh6 in the hearts of the exposed rats compared with the sham-exposed rats (Figures 4A–E).

These results indicated that cardiac remodeling was not induced by 500 μT EMF exposure at the molecular level in rats.

### Exposure to a 50-Hz EMF at 500 μT Had No Effect on Cardiac Function

**Figure 5:**
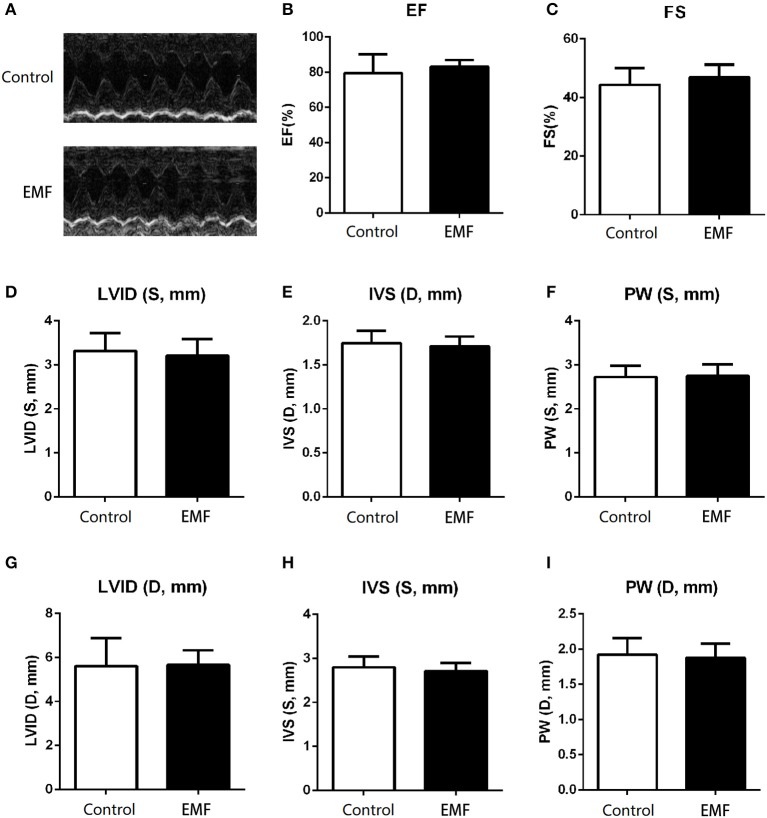
Exposure to a 50-Hz EMF at 500μT exerted no effect on cardiac function. **(A)** Echocardiography analysis of rats from the two groups. **(B)** Ejection fraction of rats from the two groups. **(C)** Fractional shortening of rats from the two groups. **(D–F)** Left ventricular internal diameter (LVID), thickness of the interventricular septum (IVS), and left ventricular posterior wall (LVPW) in the end-systole of rats from the two groups. **(G–I)** LVID, IVS, and LVPW in the end-diastole of rats from the two groups.

The main function of the heart is to pump blood throughout the body so as to meet the needs of the body for oxygen and remove carbon dioxide from the body (38). We evaluated the cardiac function of rats using echocardiography and cardiac catheterization analyses after 24 weeks of exposure. As shown in Figure 5A, the representative echocardiography images of rats from the two groups show similar manifestations. In line with this, no significant differences in the ejection fraction (EF) or fractional shortening (FS) were detected between the two groups (Figures 5B,C). In addition, the thickness of the ventricular wall was also measured. Compared to control rats, neither the left ventricular internal diameter (LVID), interventricular septum (IVS), or left ventricular posterior wall (LVPW) in either systole or diastole were affected by the 500 μT EMF exposure (Figures 5D–I).

**Figure 6:**
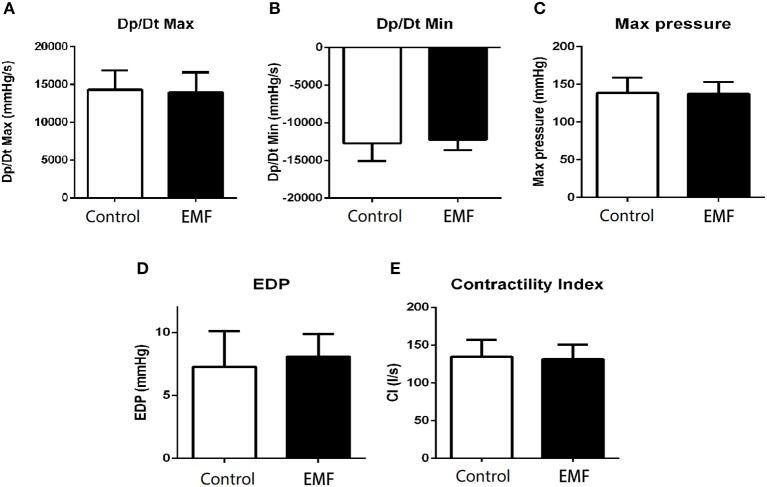
Exposure to a 50-Hz EMF at 500μT had no effect on cardiac hemodynamics. **(A)** Maximum rates of left ventricular diastolic pressure change (Dp/Dt_max_) of rats from the two groups. **(B)** Minimum rates of left ventricular diastolic pressure change (Dp/Dt_min_) of rats from the two groups. **(C)** Maximum left ventricular pressure of rats from the two groups. **(D)** Left ventricular end-diastolic pressure of rats from the two groups. **(E)** Contractility index of rats from the two groups.

Moreover, cardiac catheterization, which is the gold standard for cardiac function detection, was carried out to further explore the effects of EMF at 500 μT. As shown in Figures 6A,B compared with the control group, the results implied that exposure to a 500 μT EMF did not alter the ventricular systolic and diastolic function. Consistently, both the maximum left ventricular pressure and the end-diastolic pressure showed no difference between the exposure group and the sham exposure group (Figures 6C,D). In addition, the contractility index from the EMF exposure group did not differ from that of the control group (Figure 6E).

Taken together, these data demonstrated that the 500 μT EMF had no significant effect on cardiac function in rats.

## Discussion

The aim of the present study is to investigate the potential effects of 50-Hz EMF exposure at 500 μT on the cardiovascular system in rats. The parameters measured were heart rate, blood pressure, pulse rate, cardiac histological morphology, molecular markers associated with cardiac remodeling, and cardiac function.

Our results showed that there were no statistical differences in heart rate or blood pressure between the two groups, which is in accordance with previous studies. In 1996, Korpinen and Partanen enrolled 41 male volunteers who were subjected to 50-Hz magnetic fields from 1.4 to 6.6 μT for 1 h, and the results showed that no significant differences in blood pressure (20). In 2003, Kurokawa et al. recruited 50 healthy volunteers who were exposed to EMFs for 2 min to 12 h at frequencies ranging from 50- to 1,000-Hz with flux densities from 20 to 100 μT, and the results showed that 50-Hz EMF exposure did not affect the heart rate of the volunteers (39). This observation was further supported by Sait et al. (40). However, in 2006, Baldi et al. (41) suggested that the heart rates of all subjects in their study changed when they were exposed to the same EMFs as in Kurokawa's study. These differences may be due to discrepancies between the experimental methods.

We also explored the effect of a 50-Hz EMF at 500 μT on cardiac hypertrophy. Similarly, compared with the sham exposure group, the results showed that exposure to a 500 μT EMF did not alter the structure and general morphology of the heart. In addition, the expressions of cardiac remodeling-related genes were not changed between the two groups, a finding similar to the results of our previous study (23).

More importantly, to evaluate the effects of 50-Hz EMFs at 500 μT on cardiac function, echocardiography, and cardiac catheterization analyses were conducted. Echocardiography is mainly used to monitor alteration in the structure of the heart, while cardiac catheterization was used directly to measure the hemodynamics of the heart, an index to assess the systolic and diastolic function of the left ventricle and the left ventricular pressure. It should be noted that 500 μT EMF exposure had no effect on cardiac function compared with the control group rats, indicating that 50-Hz EMFs below 500 μT might be safe for the public.

All in all, our present study found that exposure to 500 μT EMFs had no effect on the cardiovascular system of rats, which is consistent with a previous study (42). Compared with the previous study, the flux density of the 50-Hz EMF in our exposure system was much higher, at 500 μT, which would be helpful to probe into the influence of high-intensity EMF exposure on public health.

However, there are also some defects in our study. Above all, our research mainly focused on the effects of continuous EMF exposure; further study should be performed to investigate the impact of intermittent exposure. Secondly, the flux density of the 50-Hz EMF we studied here was 500 μT, and different flux densities and frequencies should also be taken into account in further study. Thirdly, only adult male rats were included in the current study; the effects of EMF on younger, older, or female rats might be different. Finally, our present study only emphasized certain parameters related with the cardiovascular system; other parameters or other systems (e.g., the endocrine system, nervous system, and so on) might be affected by EMFs. Experiments could be conducted in animals of different ages (younger-older), gender, or condition (for example, pregnant rats) under various situations in the future.

In conclusion, our current study demonstrated that exposure to a 50-Hz EMF at 500 μT exerted no influence on the cardiovascular system in SD rats.

## Data Availability Statement

The datasets generated for this study are available on request to the corresponding author.

## Author Contributions

YZ and LL conceived and designed the experiments, performed the experiments, analyzed the data, and contributed to the writing of the manuscript. XL conceived and designed the experiments, performed the experiments, analyzed the data, and contributed the analysis tools. LD and XW conceived and designed the experiments and contributed the analysis tools. JW, MH, HH, and GR performed the animal experiments. JL and CC conceived and designed the experiments and contributed to the writing of the manuscript. All authors read and approved the final manuscript.

### Conflict of Interest

The authors declare that the research was conducted in the absence of any commercial or financial relationships that could be construed as a potential conflict of interest.
