# Supplementary material for: Examination of the Effect of a 50-Hz Electromagnetic Field at 500 μT on Parameters Related With the Cardiovascular System in Rats
Source: Front Public Health. 2020 Apr 7;8:87. doi: 10.3389/fpubh.2020.00087 (PMC7154052; doi:10.3389/fpubh.2020.00087)
Supplement: Supplementary file 1 [file Table_1.DOCX]

**Supplementary Figure 1**. The distribution of the 500 μT EMF in the rack.


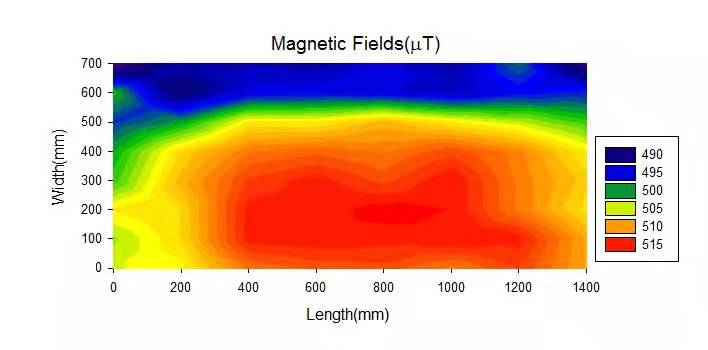


**Supplementary Table 1**. Primers of real-time PCR.

| **Primers** | **Forward 5’→ 3’** | **Reverse 5’→ 3’** |
| --- | --- | --- |
| **ANP** | GCTTCGGGGGTAGGATTGAC | TCTCAGTGGCAATGCGACC |
| **BNP** | TCCAAGATGGCACATAGTTCAAG | CAACCTCAGCCCGTCACAG |
| **MYH6** | CAGAAAATGCACGATGAGGA | GCATTCATATTTATTGTGGG |
| **GAPDH** | GGCAAGTTCAACGGCACAG | CGCCAGTAGACTCCACGACAT |
